# Supplementary material for: Plasma Proteomic Profiling Reveals the Regulatory Factors of Milk Protein Synthesis in Holstein Cows
Source: Biology (Basel). 2022 Aug 19;11(8):1239. doi: 10.3390/biology11081239 (PMC9404965; doi:10.3390/biology11081239)
Supplement: Supplementary file 1 [file biology-11-01239-s001.zip › biology-1877993-supplementary.pdf]

# Plasma Proteomic Profiling Reveals the Regulatory Factors of Milk Protein Synthesis in Holstein Cows

Xinling Wang [1], Jie Xu [1] and Zhaoyu Han [1, *]

[1] Affiliation 1; College of Animal Science and Technology, Nanjing Agricultural University, Nanjing 210095, China
*Correspondence: zyhan6708@njau.edu.cn ; Tel: +86-25-84395314; Fax: +86-25-84395314

**A**

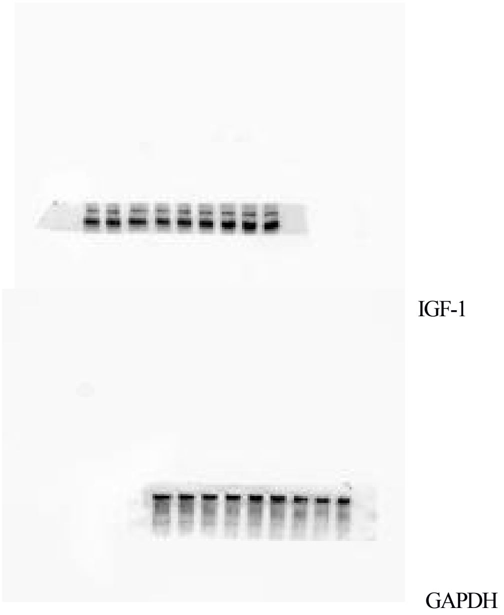


| Group | Sample | IGF-1 |
|---|---|---|
| | L-1 | 0.936 |
| LMP | L-2 | 0.823 |
| | L-3 | 0.728 |
| | M-1 | 0.896 |
| MMP | M-2 | 1.137 |
| | M-3 | 0.967 |
| | H-1 | 0.947 |
| HMP | H-2 | 1.437 |
| | H-3 | 1.570 |

**B**

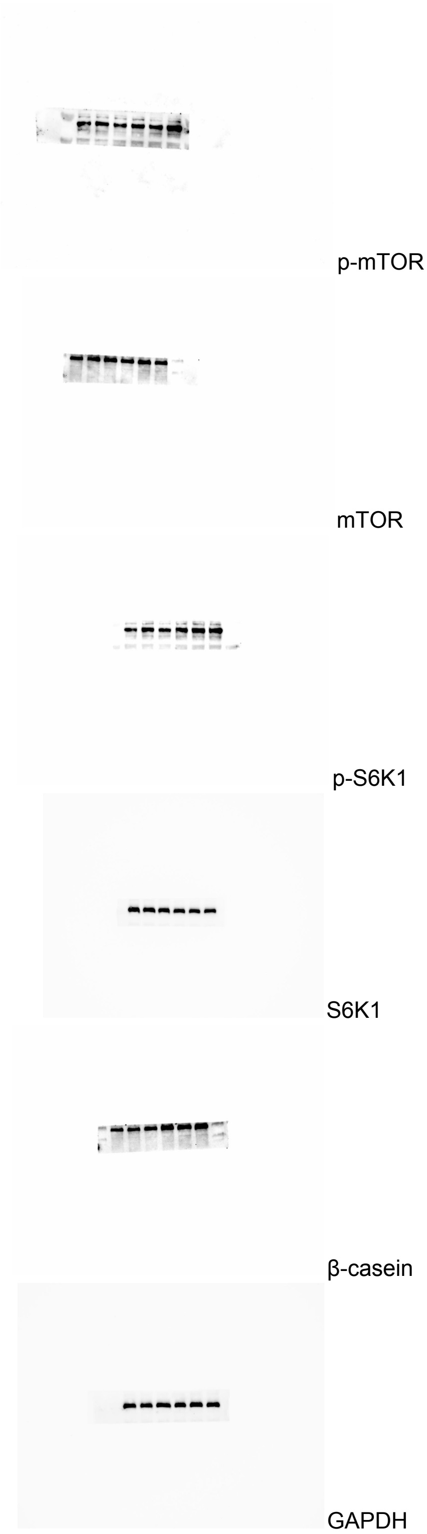


| Group | Sample | Protein | | | | |
|---|---|---|---|---|---|---|
| | | β-casein | p-mTOR | mTOR | p-S6K1 | S6K1 |
| Control | Con-1 | 0.908 | 0.978 | 0.992 | 1.004 | 1.095 |
| | Con-2 | 0.951 | 1.014 | 0.989 | 1.154 | 1.030 |
| | Con-3 | 1.141 | 0.804 | 1.018 | 1.047 | 1.087 |
| IGF-1 | IGF1-1 | 1.587 | 1.392 | 1.003 | 1.340 | 0.937 |
| | IGF1-2 | 1.561 | 1.339 | 1.105 | 1.364 | 0.985 |
| | IGF1-3 | 1.499 | 1.573 | 1.096 | 1.388 | 0.918 |

**Figure S1**. Original data of western blot and the densitometry reading ratio of each band in (**A**) Figure 4A and (**B**) Figure 5B.
